# Supplementary material for: Proper chromosome alignment depends on BRCA2 phosphorylation by PLK1
Source: Nat Commun. 2020 Apr 14;11:1819. doi: 10.1038/s41467-020-15689-9 (PMC7156385; doi:10.1038/s41467-020-15689-9)
Supplement: Supplementary file 1 — Supplementary Information [file 41467_2020_15689_MOESM1_ESM.pdf]

Supplementary information

Ehlen *et al.*,

Supplementary Fig. 1

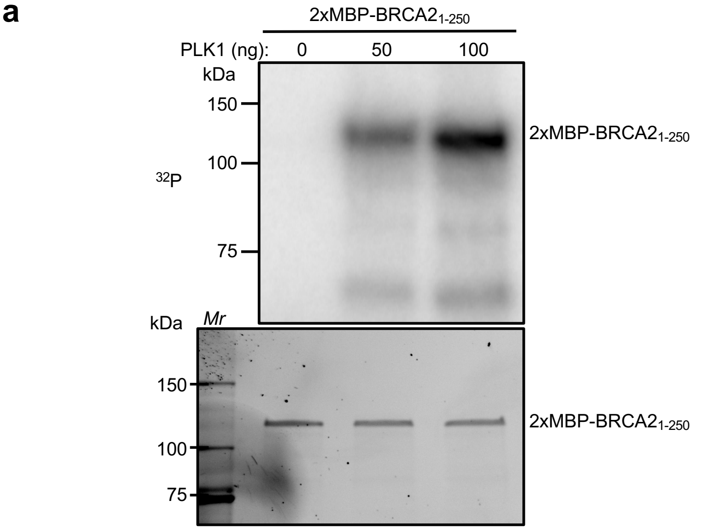

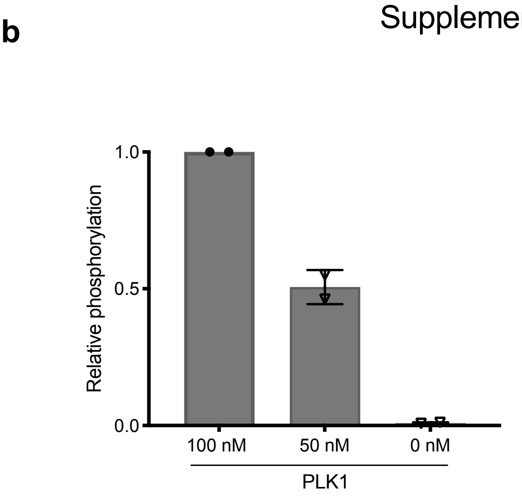

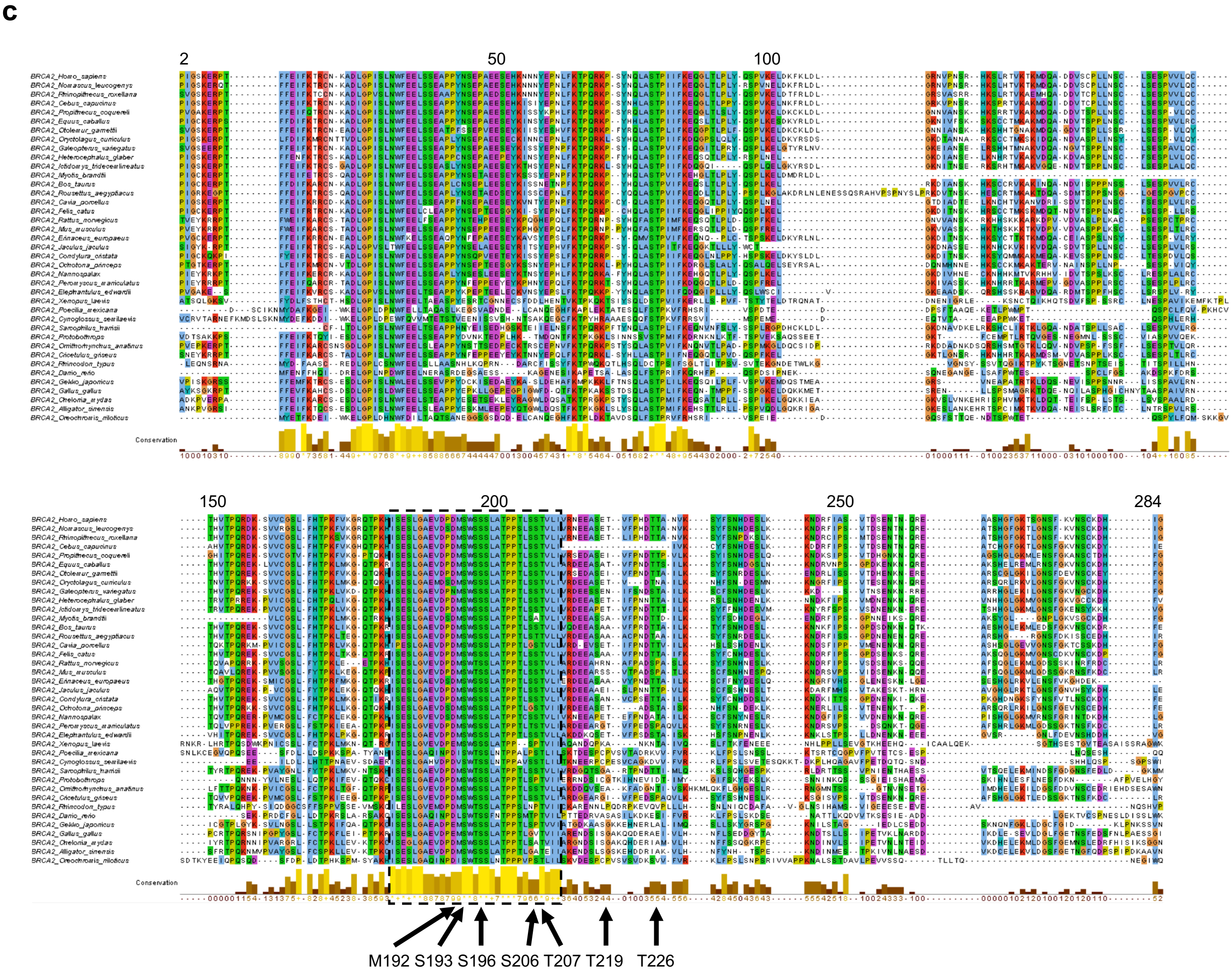

Supplementary Figure 1. Related to Figure 1. **PLK1 phosphorylation of the N-terminal region of BRCA2 and conservation of PLK1 phosphosites**

(a) PLK1 *in vitro* kinase assay with $BRCA2_{1-250}$. Top: The polypeptide 2x-MBP-$BRCA2_{1-250}$ WT was incubated with increased concentrations (0, 50 and 100 ng) of recombinant PLK1 in the presence of γ^{32}P-ATP. The samples were resolved on 4-15% SDS-PAGE and the ^{32}P-labeled products were detected by autoradiography. Bottom: 4-15% SDS-PAGE showing the input of 2xMBP-$BRCA2_{1-250}$ WT (0.5 μg) used in the reaction. **(b)** Quantification of the relative phosphorylation in (a). Data are represented as mean ± SD from two independent experiments. **(c)** Alignment of the region 2-284 of human BRCA2 with the N-terminal regions of BRCA2 from 40 different species. Amino acids conserved in more than 30 % of the species are highlighted with coloured background. A dashed line box identifies the highly conserved cluster around S193 (amino acid 180 to amino acid 210). Arrows show the amino acids cited in this manuscript (including the PLK1 phosphosites). Source data is available as a Source Data file.

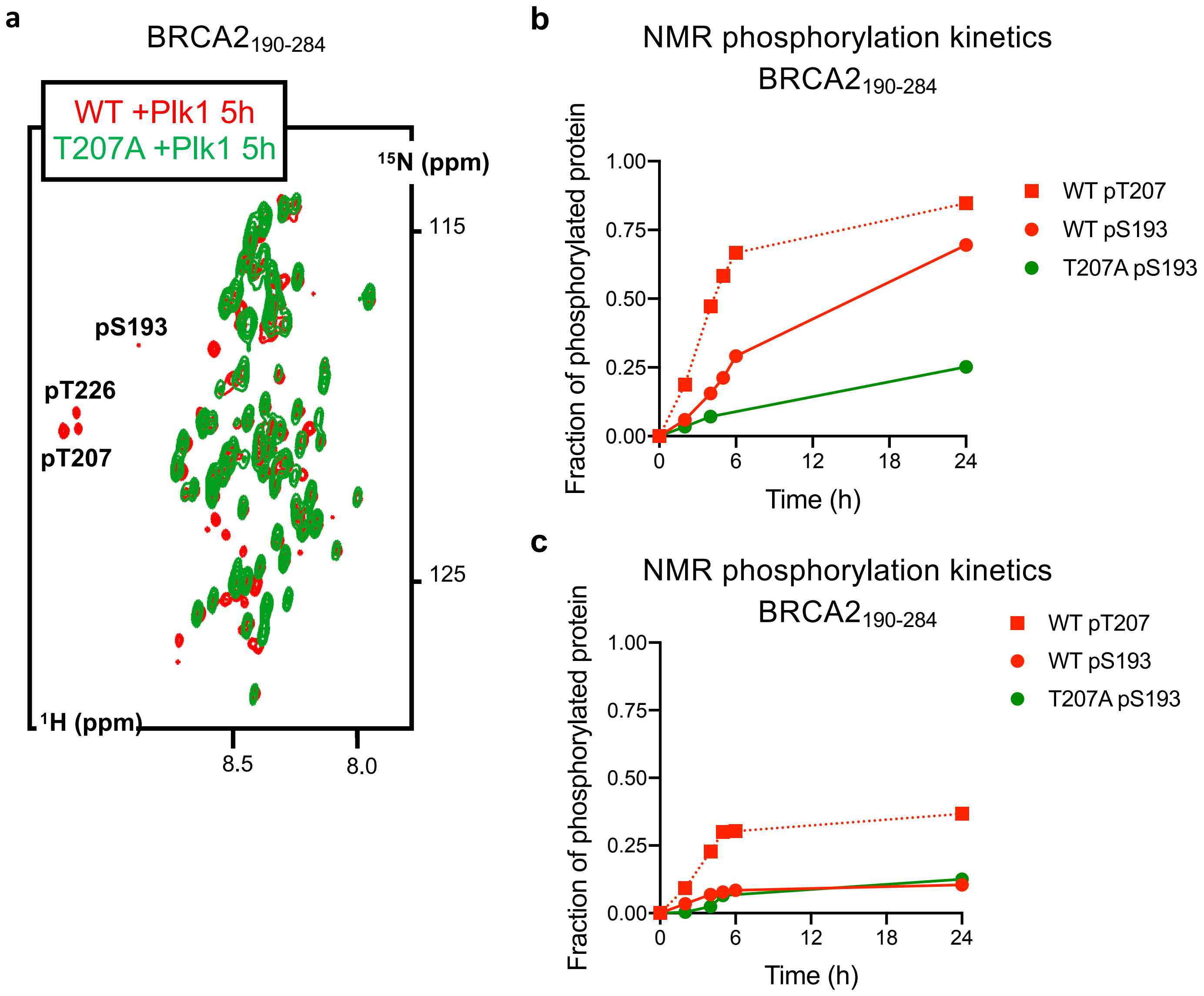
a
BRCA2190-284
WT +Plk1 5h
T207A +Plk1 5h
15N (ppm)
115
125
pS193
pT226
pT207
1H (ppm)
8.5
8.0
b
NMR phosphorylation kinetics
BRCA2190-284
Fraction of phosphorylated protein
1.00
0.75
0.50
0.25
0.00
0
6
12
18
24
Time (h)
WT pT207
WT pS193
T207A pS193
c
NMR phosphorylation kinetics
BRCA2190-284
Fraction of phosphorylated protein
1.00
0.75
0.50
0.25
0.00
0
6
12
18
24
Time (h)
WT pT207
WT pS193
T207A pS193

Supplementary Figure 2. Related to Figure 2. **PLK1 phosphorylation kinetics of $BRCA2_{190-284}$ WT vs T207A**

(a) Superimposition of the ^{1}H-^{15}N HSQC spectra recorded on the ^{15}N labelled fragments $BRCA2_{190-284}$ WT (red) and T207A (green) after 5h of incubation with PLK1. The conditions are the same as in Figure 1. **(b,c)** Comparison of the phosphorylation kinetics of $BRCA2_{190-284}$ WT and T207A performed with two different PLK1 samples. The percentage of phosphorylation deduced from the intensities of the peaks corresponding to the non-phosphorylated and phosphorylated residues is plotted as a function of time. WT S193 and T207 time points are represented by red circles and squares, respectively, while T207A S193 timepoints are represented by green circles. Source data is available as a Source Data file.

Supplementary Fig. 3

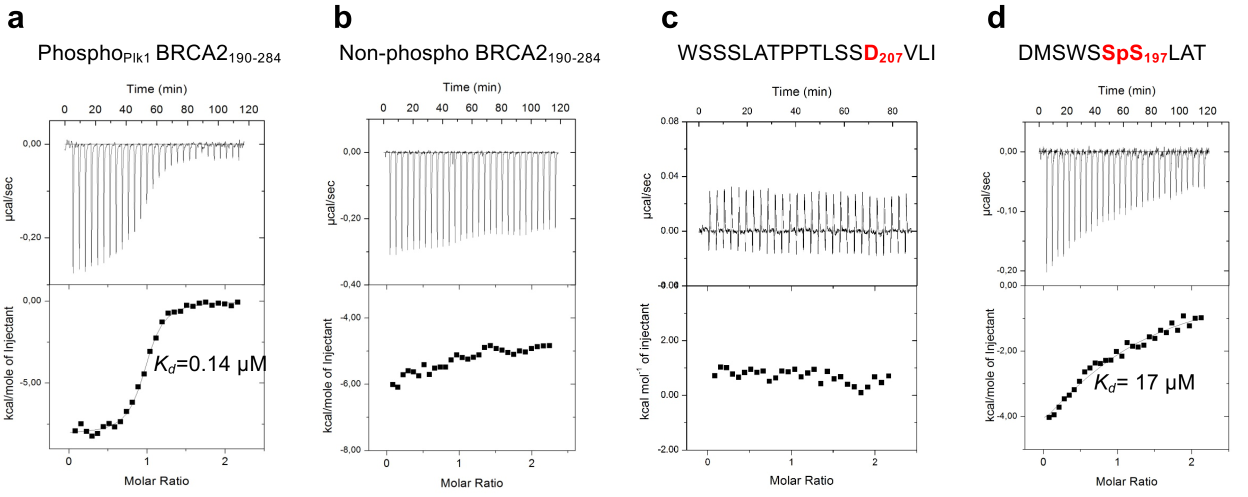

Supplementary Figure 3. Related to Figure 3. **Isothermal Titration Calorimetry (ITC) thermogram showing binding of $PLK1_{PBD}$ to the fragment $BRCA2_{190-284}$ or a 10 aa BRCA2 peptide containing pS197**

Thermogram showing the binding affinity of $PLK1_{PBD}$ to the **(a)** phosphorylated or **(b)** non-phosphorylated $BRCA2_{190-284}$ fragment, purified from bacteria as explained in the Methods section and also used in the NMR experiments. **(c)** Thermogram showing the binding affinity of $PLK1_{PBD}$ to a 17 aa BRCA2 synthetic peptide comprising T207D ($WSSSLATPPTLSSD_{207}VLI$). **(d)** Thermogram showing the binding affinity of $PLK1_{PBD}$ to a 10 aa BRCA2 synthetic peptide comprising pS197. Source data is available as a Source Data file.

Supplementary Fig. 4

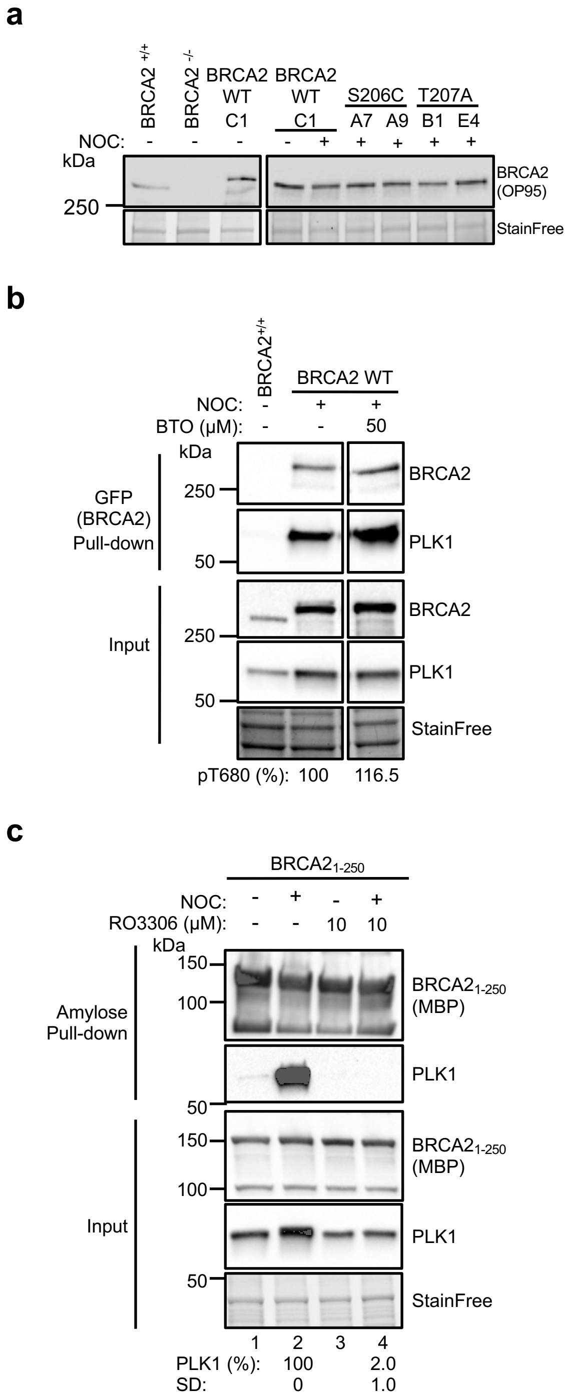

Supplementary Figure 4. Related to Figures 3-8**. BRCA2 protein levels in DLD1 BRCA2$^{-/-}$ stable clones bearing BRCA2 WT and variants utilized in this study and effect of PLK1 and CDK1 inhibitors on the interaction between BRCA2 and PLK1**

(a) BRCA2 protein levels in total cell extracts from DLD1 cells stably expressing EGFP-MBP-BRCA2 WT (BRCA2 WT C1) or the variants S206C (clones A7 and A9) and T207A (clones B1 and E4) as detected by western blot using anti-BRCA2 (OP95) antibody. **(b)** The effect of PLK1 inhibitors on the interaction between BRCA2 WT and PLK1. BRCA2 WT cells were treated with nocodazole (100 ng/ml, 14h), then treated with the PLK1 inhibitor BTO (50 μM, 2h) before harvesting. BRCA2 was pull-down with GFP-trap beads, immunocomplexes were resolved on 4-15% SDS-PAGE followed by WB using anti-PLK1 and -MBP antibodies. Unsynchronized BRCA2$^{+/+}$ were used as control of pull-down and StainFree images of the gels before transfer were used as loading control (cropped image is shown). The amount of PLK1 co-IPed with BRCA2 relative to the input levels of PLK1 and the amount of pull-down BRCA2 is presented below the blot, relative to non-treated BRCA2 WT. **(c)** The effect of CDK1 inhibitors on the interaction between 2xMBP-BRCA2$_{1-250}$ and PLK1. U2OS cells transiently transfected with the 2xMBP-BRCA2$_{1-250}$ WT, were arrested in mitosis with nocodazole (300 ng/ml, 14h) and then treated with CDK1 inhibitor (Ro-3306 50nM, 2h) before amylse pull-down. Complexes were resolved on 4-15% SDS-PAGE followed by WB using anti-PLK1 and anti-MBP antibodies. The amount of PLK1 co-immunoprecipitated with 2xMBP-BRCA2$_{1-250}$ relative to the input levels of PLK1 and the amount of pull-down 2xMBP-BRCA2$_{1-250}$ is presented below the blot as mean ± SD from three independent experiments. The data is presented relative to the non-treated BRCA2 WT. Imunoblots in (a) and (c) are representative of three independent experiments. Source data is available as a Source Data file.

Supplementary Fig 5

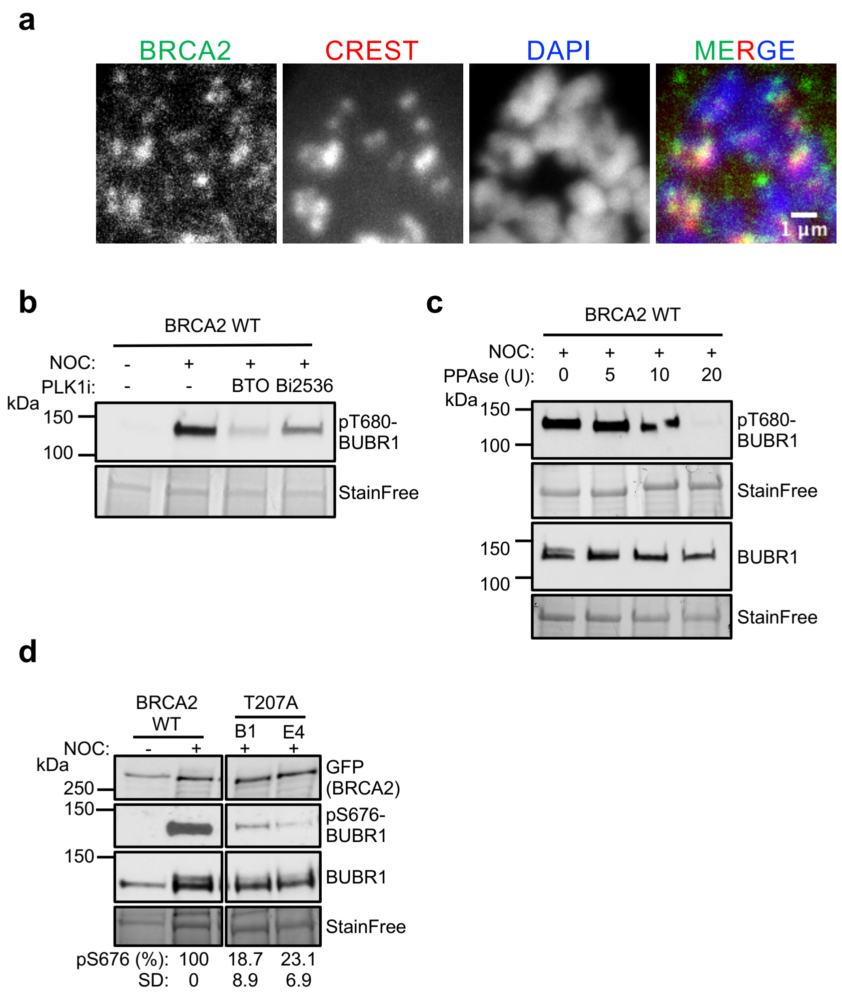

Supplementary Figure 5. Related to Figure 5**. BRCA2 at the kinetochores and effect of PLK1 inhibitors and phosphatase treatment on pT680-BUBR1.**

(a) Representative images of the localization of BRCA2 in nocodazole-arrested U2OS transient expressing GFP-MBP-BRCA2 WT. BRCA2 is detected by anti-BRCA2 rabbit antibody (CA1033), CREST is used as centromere marker and DNA is counterstained with DAPI. Scale bar represents 1 µm. The figures are representative of three independent experiments**. (b)** Protein levels of pT680-BUBR1 in BRCA2 WT stable clone after treatment with PLK1 inhibitors. After 14h culture with media containing nocodazole (100 ng/µl), PLK1 inhibitors (Bi2536 (50 nM) or BTO (50 µM)) were added to the media and the cells were cultured for additional 2h before harvesting. The level of pT680-BUBR1 was analyzed in the total protein extract by western blot. **(c)** Phosphatase (Fast AP) treatment of total protein lysate extracted from DLD1 BRCA2 WT cells treated with nocodazole (100 ng/µl) for 14h. **(d)** Western blot showing the expression levels of endogenous BUBR1 and pS676-BUBR1 in nocodazole treated stable clones of DLD1 BRCA2 deficient cells ($BRCA2^{-/-}$) expressing GFPMBP-BRCA2 WT (BRCA2 WT) or T207A variant. The levels of pS676-BUBR1 was analyzed in the total protein extract by western blot. The mean pBUBR1 signal relative to the stain free signal is shown for the nocadozole treated samples below the blots. Results are presented normalized to the protein levels for BRCA2 WT. The data represents the mean ± SD of two independent experiments. Imunoblots in (b-d) are representative of two (b, d) and three (c) independent experiments. Source data is available as a Source Data file.

Supplementary Fig. 6

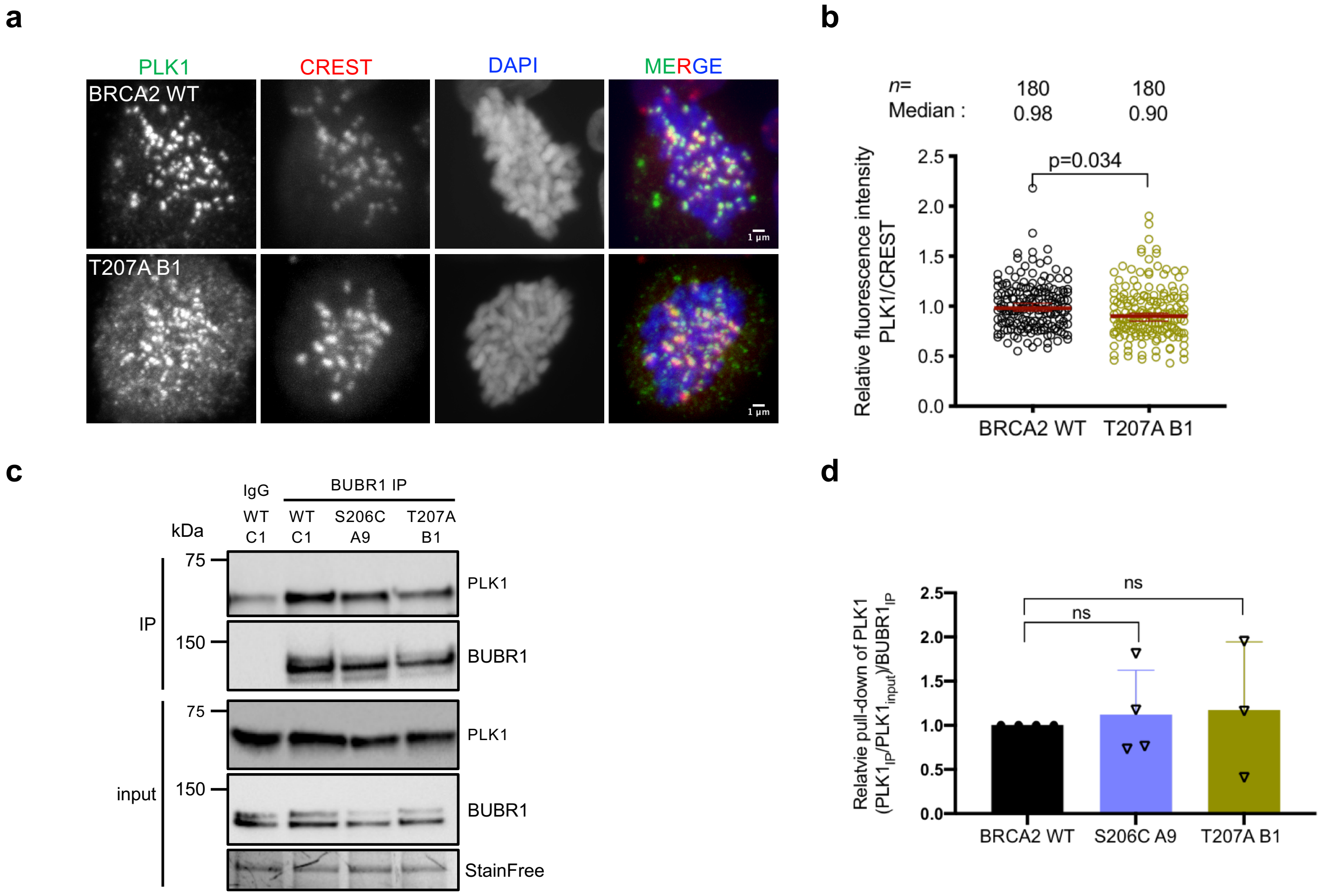

Supplementary Figure 6. Related to Figure 5. **Localization of BRCA2 and PLK1 at the kinetochores and PLK1-BUBR1 complex in cells bearing variant T207A compared to WT**

(a) Representative images of the localization of PLK1 in nocodazole-arrested DLD1 BRCA2$^{-/-}$ cells stably expressing GFP-MBP-BRCA2 WT or the variant T207A as indicated. CREST is used as centromere marker and DNA is counterstained with DAPI. Scale bar represents 1 µm. **(b)** Quantification of the co-localization of PLK1 and CREST in (a). The data represents the intensity ratio (PLK1:CREST) relative to the mean ratio of PLK1:CREST for the GFP-MBP-BRCA2 WT calculated from a total of 180 pairs of chromosomes analysed from two independent experiments (6 pairs of chromosomes/cell from 15 cells). The red line in the plot indicates the median (95% CI) ratio, each dot represents a pair of chromosomes. For statistical comparison of the differences between the samples we applied a Mann-Whitney two-tailed analysis, the p-values show significant difference. **(c)** Co-immunoprecipitation of endogenous PLK1 with endogenous BUBR1 from mitotic cell extracts of BRCA2 WT cells or cells expressing the variants S206C or T207A using mouse anti-BUBR1 antibody. Mouse IgG was used as control for the BUBR1 immunoprecipitation. The immuno-complexes were resolved on 4-15% SDS-PAGE followed by western blotting, the interactions were revealed by rabbit anti-BUBR1 and mouse anti-PLK1 antibodies. **(d)** Quantification of co-immunoprecipitated PLK1 in (c), relative to the input levels and the amount of immunoprecipitated BUBR1. Results are presented as the fold change compared to the BRCA2 WT clone. The data represents the mean ± SD of three to four independent experiments (WT (n=4), S206C A9 (n=4) and T207A B1 (n=3)). Statistical significance of the difference was calculated with one-way ANOVA test with Dunnett's multiple comparisons test, the p-values show the significant difference, ns: non-significant. Source data is available as a Source Data file.

Supplementary Fig. 7

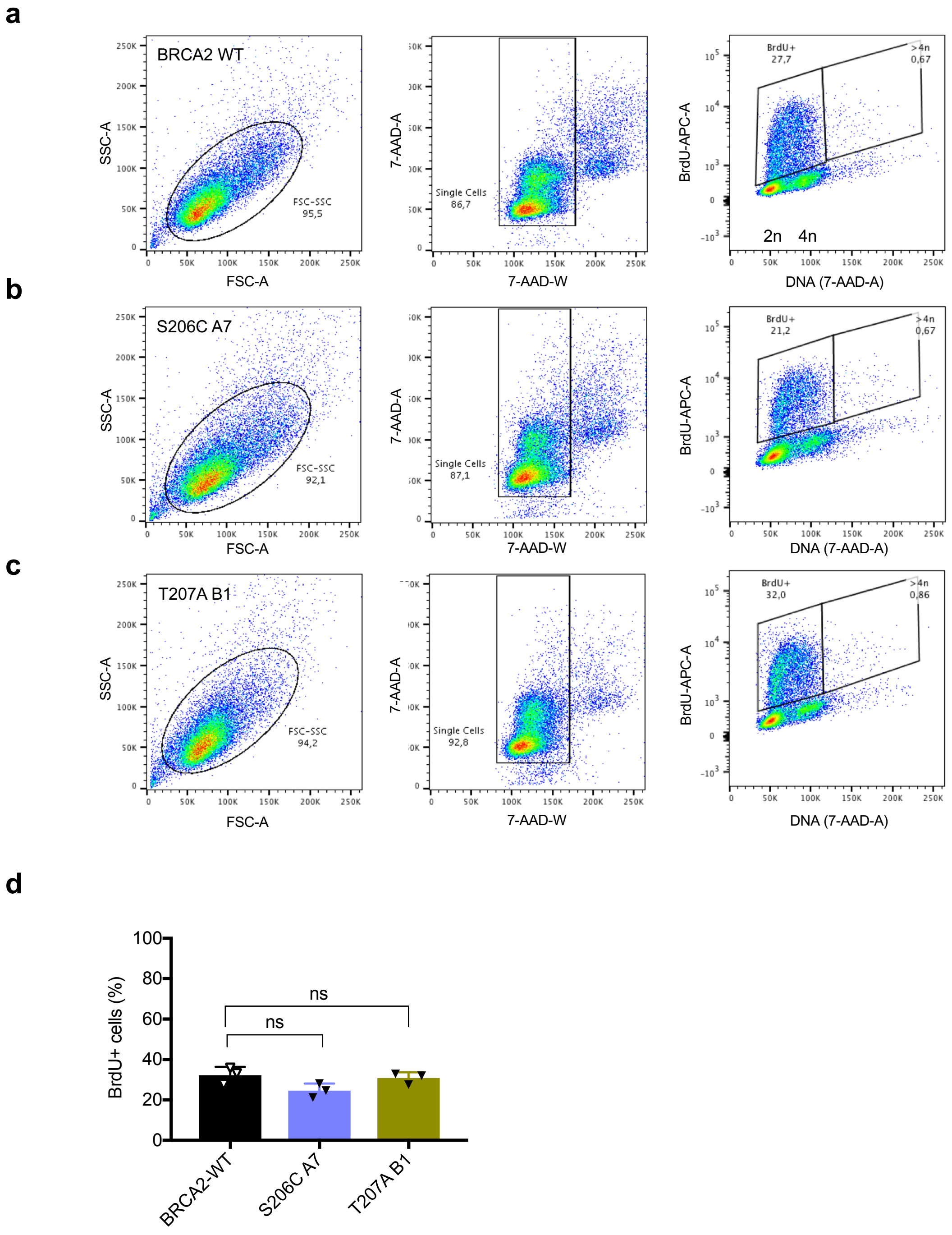

Supplementary Figure 7. Related to Figure 7**. BrdU incorporation measured by flow cytometry of DLD1 BRCA2$^{-/-}$ stable cell lines expressing BRCA2 WT or BRCA2 variants S206C and T207A.**

(a-c) Representative flow cytometry plots for the analysis of S-phase tetraploid cells (quantified in Figure 7c, d) in the stable DLD1 BRCA2 deficient cells expressing BRCA2 WT (a) or the VUS S206C (b) and T207A (c). Viable cells were gated from the Forward Scatter (FSC-A) versus Side Scatter (SSC-A) plots and displayed in a 7-AAD-W versus 7-AAD-A plot to exclude doublets. The gated singlet population was displayed in a APC-A (BrdU) versus 7-AAD-A (DNA) plot. The S-phase tetraploid population was gated as BrdU^{+} cells with DNA content >4N. 20,000 singlet events were collected for each experiment. **(d)** Frequency of BrdU^{+} cells in the stable clones expressing BRCA2 WT or the VUS as indicated. The data represents the mean ± SD of three independent experiments. Statistical significance of the difference was calculated with one-way ANOVA test with Tukey's multiple comparisons test (the p value show the difference compared to WT, ns: non-significant). Source data is available as a Source Data file.

Supplementary Fig. 8

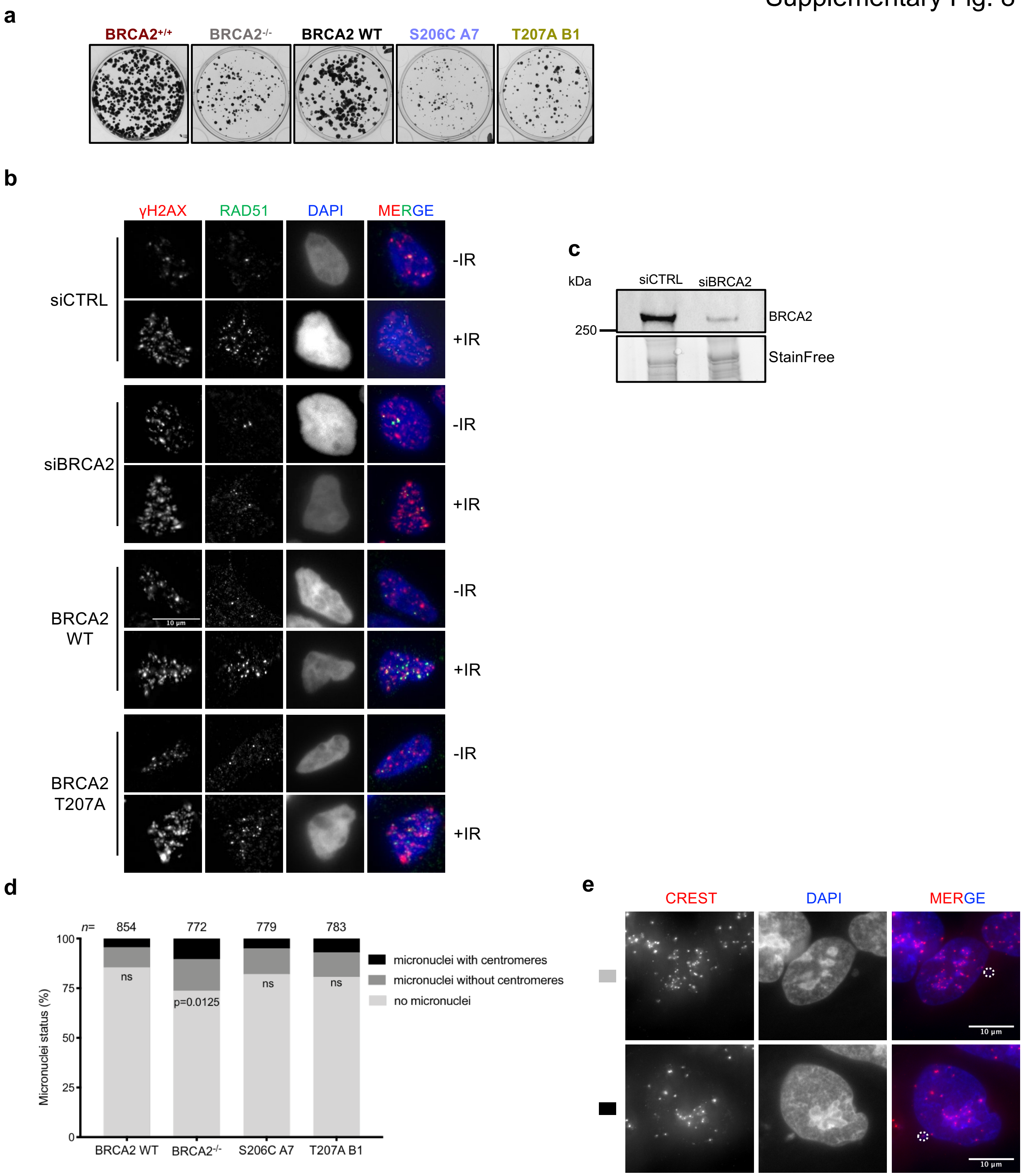

Supplementary Figure 8. Related to Figure 8**. Plating efficiency of unchallenged DLD1 BRCA2$^{-/-}$ stable clones expressing EGFP-MBP-BRCA2 WT or the variants. and representative images of DNA damage foci in these cells. Micronuclei in BRCA2$^{-/-}$ stable cell lines and BRCA2$^{-/-}$ expressing BRCA2 WT or BRCA2 variants S206C and T207A.** **(a)** Representative plates showing the number of colonies in unchallenged conditions of the cells assessed for MMC-clonogenic survival assay quantified in Fig. 9a (500 cells seeded per 6-well plate). **(b)** Representative immunofluorescence images of two independent experiments of nuclear γH2AX and RAD51 foci in DLD1 BRCA2$^{+/+}$ cells depleted of BRCA2 (siBRCA2), BRCA2$^{+/+}$control cells (siCTRL), DLD1 BRCA2 deficient cells (BRCA2$^{-/-}$) stably expressing BRCA2 WT or the variant T207A, in non-treated (-IR) or two hours after exposure to 6 Gy of γ-irradiation (+IR), as indicated in the images and as quantified in Fig. 9d, 9e. Scale bar represents 10 μm. **(c)** Representative Western blot of two independent experiments showing the levels of endogenous BRCA2 in the siRNA transfected cells imaged in (b) and analysed in Fig. 9d, 9e, at the time for radiation. **(d)** Frequency of micronuclei with and without centromeres in DLD1 BRCA2 deficient cells (BRCA2$^{-/-}$) and BRCA2$^{-/-}$ clones stably expressing BRCA2 WT or the variants S206C and T207A. *n* indicates the total number of cells counted for each clone from two independent experiments. Statistical significance of the difference was calculated with two-way ANOVA test with Tukey's multiple comparisons test, the p-values show the significant difference, ns: non-significant. **(e)** Representative images of micronuclei with and without centromeres observed in cells quantified in (d), scale bar represents 10 μm. Source data is available as a Source Data file.

Supplementary Fig. 9

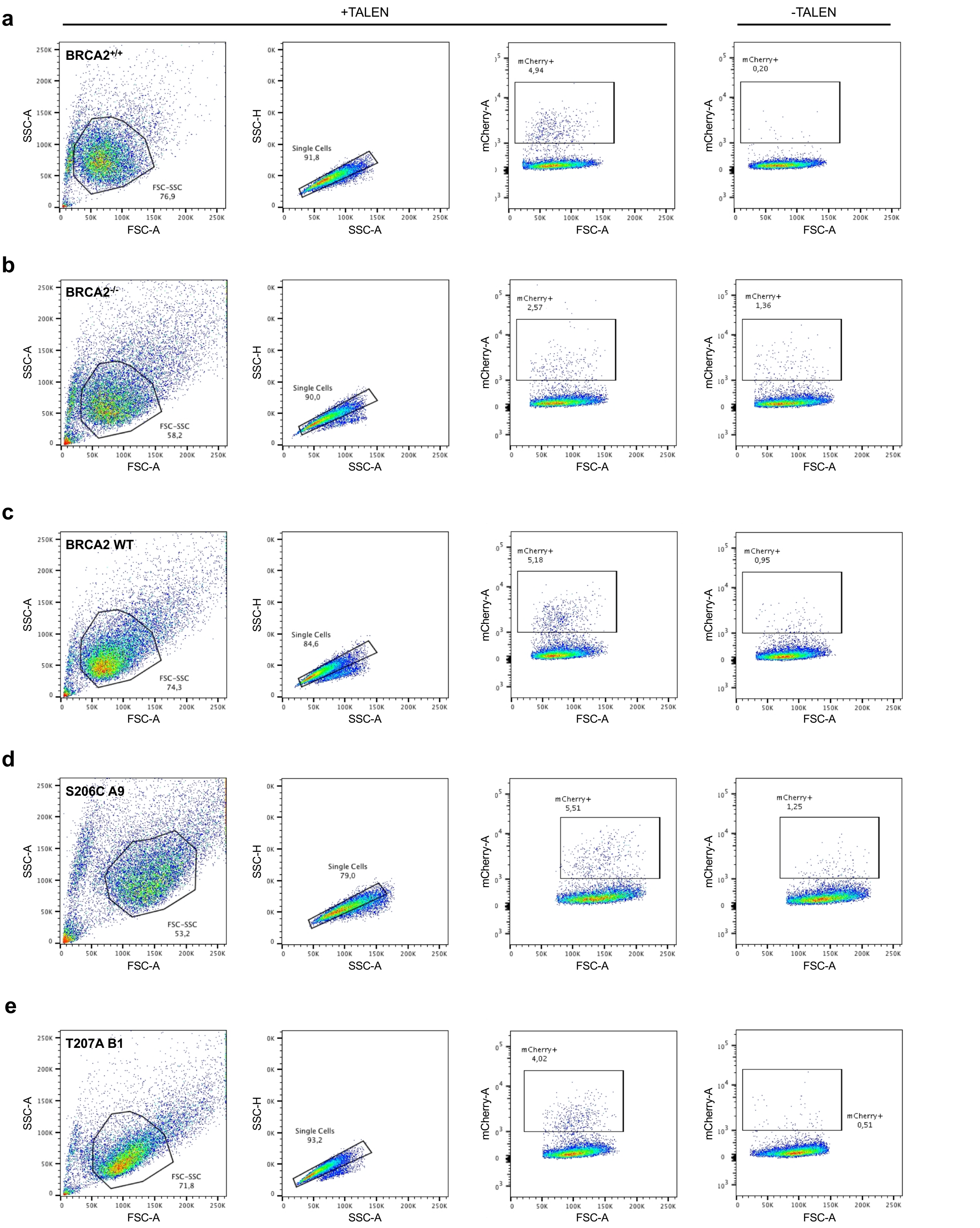

Supplementary Figure 9. Related to Figure 8f**. Frequency of mCherry positive cells measured by flow cytometry of DLD1 BRCA2$^{-/-}$ stable cell lines expressing BRCA2 WT or BRCA2 variants S206C and T207A.**

(a-e) Representative flow cytometry plots for the analysis of mCherry positive cells in the HR assay (quantified in Fig. 9f) in DLD1 cells with endogenous BRCA2 (BRCA2$^{+/+}$) (a), DLD1 BRCA2 deficient cells (BRCA2$^{-/-}$) (b), BRCA2 deficient cells expressing BRCA2 WT (c) or the VUS S206C (d) and T207A (e). Viable cells were gated from the Forward Scatter (FSC-A) versus Side Scatter (SSC-A) plots and displayed in a SSC-H versus SSC-A plot to exclude doublets. The gated singlet population was displayed in a mCherry-A versus FSC-A plot. The mCherry positive population was gated from non-transfected cells. 10 000 singlet events were collected for each experiment. Source data is available as a Source Data file.

Supplementary Figure 10

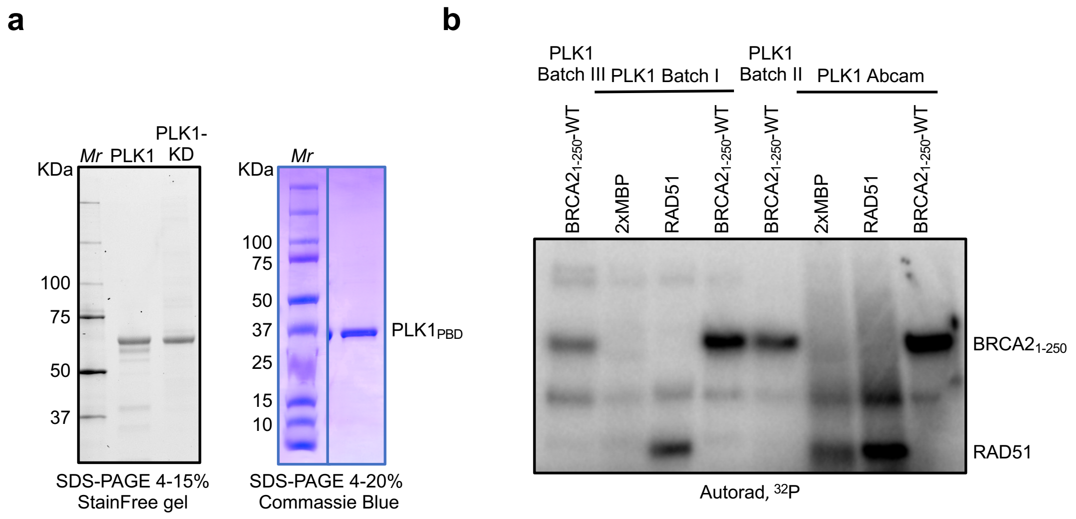

Supplementary Figure 10. SDS-PAGE of the PLK1, PLK1-KD and PLK1-PBD recombinant proteins utilized in this study and comparison of the kinase activity of each batch of PLK1

(a) SDS-PAGE showing purified PLK1, PLK1-K82R mutant (PLK1-KD) and $PLK1_{PBD}$. Human PLK1 was expressed and purified from sf9 insect cells using Ni-NTA column followed by a second purification step with a cationic exchange Capto S column. Purified PLK1 and PLK1-K82R protein (3 µg) were loaded on a 4-15% SDS-PAGE Stain-Free gel. For purification of $PLK1_{PBD}$, 6His-Sumo-$PLK1_{PBD}$ was expressed and purified from bacteria using a His-TRAP column, the His-tag was cleaved with 6xHis-SUMO Protease and the cleaved $PLK1_{PBD}$ was further purified using Ni-NTA agarose resin. The purified protein was loaded on a 4-20% SDS-PAGE (1.4 µg) and detected by Coomassie staining. *Mr;* molecular weight markers. **(b)** *In vitro* kinase assay with the purified PLK1 (0.1 µg) from (a) or PLK1 purchased from Abcam, 0.1 µg PLK1 was used in the kinase reaction with either RAD51 (25 ng) or purified 2xMBP-$BRCA2_{1-250}$ WT (0.5 µg) as substrate in the presence of [γ^{32}P]-ATP. The samples were resolved by 7.5 % SDS-PAGE and ^{32}P-labeled products were detected by autoradiography. Source data is available as a Source Data file.

Supplementary Table 1: Number of records in ClinVar and BRCAShare data bases of the variants identified in breast cancer patients altering the amino acids investigated in this work. The specific VUS utilized in this study are highlighted in bold.

VUS	ClinVar	BRCAShare
**M192T**	2	0
S196I	4	0
**S196N**	4	3
S196T	1	0
**S206C**	1	1
S206Y	1	0
**T207A**	3	0
T207I	4	0

Supplementary Table 2: Statistics table for the crystal structure of PBD_pT207, a complex between the Polo-Box Domain of human PLK1 (aa 365 to aa 603) and the 17aa peptide pT207 of BRCA2 (aa 194 to aa 210, threonine 207 being phosphorylated).

	PBD_T207 phosphorylated (PDB 6GY2)
**Data collection**	
Space group	P_1
Cell dimensions :	
a, *b*, *c* (Å)	50.000 56.040 61.030
α, β, γ (°)	80.79 79.23 65.05
Molecules per a.u	2
Resolution (Å)	59.70 – 3.106 (3.106 – 3.16)
R_{merge}	0.056 (0.346)
R_{meas}	0.076 (0.466)
R_{pim}	0.051 (0.31)
$I/\sigma(I)$	10.1 (2.3)
$CC_{1/2}$	0.997 (0.821)
Completeness (spherical, %)	93.7 (96.9)
Redundancy	1.94 (1.977)
B Wilson (Å^2)	76.9 (78.86)
Multiplicity	1.9 (2.0)

**Refinement**	
Resolution (Å)	22.57 - 3.11 (3.11 – 3.15)
No. reflections	9911
R_{work} / R_{free}	0.189/0.215
No. Atoms	
Protein	3788
Heterogen atoms	28
Water	22
R.m.s. deviations	
Bond lengths (Å)	0.007
Bond angles (°)	0.97

*Values in parentheses are for highest-resolution shell.

Supplementary Table 3: Primers used to introduce point mutations in EGFPMBP-BRCA2, 2xMBP-BRCA2$_{1-250}$, GST-BRCA2$_{190-284}$ constructs

Mutation	Oligo name	Sequence (5'-3')
S193A	Fw : oAC543	CCC ACC CTT AGT TCT GCT GTG CTC ATA GTC
	Rv : oAC544	GAC TAT GAG CAC AGC AGA ACT AAG GGT GGG
M192T	Fw : oAC283	GTGGATCCTGATACGTCTTGGTCAAGTTC
	Rv : oAC284	GA ACT TGA CCA AGA CGT ATC AGG ATC CAC
S196N	Fw : oAC026	CCTGATATGTCTTGGTCAAATTCTTTAGCTACACCACC
	Rv : oAC027	GGTGGTGTAGCTAAAGAATTTGACCAAGACATATCAGG
S206C	Fw : oAC028	CCACCCACCCTTAGTTGTACTGTGCTCATAGTCAG
	Rv : oAC029	CTGACTATGAGCACAGTACAACTAAGGGTGGGTGG
T207A	Fw : oAC545	GGA TCC TGA TAT GGC TTG GTC AAG TTC TTT AGC

	Rv : oAC546	GCT AAA GAA CTT GAC CAA GCC ATA TCA GGA TCC

Supplementary Table 4: Sequencing primers

Construct	Oligo name	Binding site	Sequence (5'-3')
GFPMBP-BRCA2, GST-BRCA2$_{190-284}$	Rv : oAC131	aa 273 BRCA2	TTAGTTCGACTTATCCAATGTGGTCTTT
2xMBP-BRCA2$_{1-250}$	Fw : oAC149	aa 1-6 BRCA2	TTATTTGCTAGCCCTATTGGATCCAAAGAG
PLK1	Fw : oAC907	aa 38 PLK1	AAAGAGATCCCGGAGGTCCTAGTG

Supplementary Table 5: Primers used to subclone BRCA2$_{190-284}$ into the pGEX-6P-1 vector

Construct	Oligo name	Sequence (5'-3')
BRCA2$_{190-284}$	Fw: oAC130	TTAGGATCCATGTCTTGGTCAAGTTCT
	Rv: oAC131	TTAGTTCGACTTATCCAATGTGGTCTTT

Supplementary Table 6: Primers used to subclone *PLK1* cDNA into pFastBac HT

Primer name	Sequence (5'-3')
GA_pFBtev_R	GCCCTGAAAATACAGGTTTTCGGTCGTTGGGAT

GA_pFB_UTR _F	TTGTCGAGAAGTACTAGAGGATCATAATCA
GA_hPLK_F	ATCCCAACGACCGAAAACCTGTATTTTCAGGGCATGAGT GCTGCAGTGACTGCA
GA_hPLK_R	TGATTATGATCCTCTAGTACTTCTCGACAATTAGGAGGC CTTGAGACGGTT

Supplementary Table 7: Primers used to introduce K82R point mutation in pFastBAC-PLK1 vector to produce PLK1-KD and to subclone $PLK1_{PBD}$ (aa 326-603) into pT7-His6-SUMO

Product	Oligo name	Sequence (5'-3')
K82R-PLK1	Fw : oAC905	GCG GGCAGGATTGTGCCTAAG
	Rv : oAC906	CTTAGGCACAATCCTGCCCGC
$PLK1_{PBD}$	GA_PLKPDBwt_F	ATTGAGGCTCACCGCGAACAGATTGGTGGCTC GATTGCTCCCAGCAGCCT
	GA_PLKPDBwt_R	TTCCTTTCGGGCTTTGTTAGCAGCCGGTCATTA GGAGGCCTTGAGACGGT

Supplementary Table 8: Primers used to introduce S670D, S676D and T680D point mutations in pcDNA3-3xFlagBUBR1-RFP construct

Name	Sequence (5'-3')
oAC884	CAA GAA GCT GGA CCC AAT TAT TGA AGA CGA TCG TGA AGC CGA CCA CTC CTC
oAC885	GAG GAG TGG TCG GCT TCA CGA TCG TCT TCA ATA ATT GGG TCC AGC TTC TTG

Supplementary Table 9: Synthetic peptide sequences for Isothermal Titration Calorimetry (ITC) and X-ray crystallography

Peptide	Sequence
pS197	DMSWSS{pS}LAT
T207	WSSSLATPPTLSSTVLI
pT207	WSSSLATPPTLSS{pT}VLI
T207A	WSSSLATPPTLSSAVLI
T207D	WSSSLATPPTLSSDVLI
CpT207	WSSSLATPPTLSC{pT}VLI

Supplementary Table 10: Primers for amplifying BRCA2 (aa 1-267) from genomic DNA

Primer name	Sequence (5'-3')
Fw : oAC035	GGTCGTCAGACTGTCGATGAAGCC
Rv : oAC056	CAAAGAGAAGCTGCAAGTCATGGATTTGAAAAAACATC AGGG

Supplementary Table 11: Oligos used for replacing GFP in AAVS1-2A-GFP to mCherry using Gibson Assembly strategy

Construct	Oligo name	Sequence (5'-3')
AAVS1-2A	Fw: oAC537	TAAAGCGGCCGCGTCGAGTCTAGAGGG
	Rv: oAC538	CATCTCGAGCCTAGGGCCGGG
AAVS1-2A-mCherry	Fw: oAC539	cccggccctaggctcgagatgGTGAGCAAGGGCGA GGAGGATAAC

	Rv: oAC540	ctctagactcgacgcggccgctttaCTTGTACAGCTCGT CCATGCCGC

Supplementary Table 12: TALEN sequences used in DSB-mediated gene targeting assay

Name	Sequence (5'-3')
TALEN AAVS 5'	TCCCCTCCACCCCACAGT
TALEN AAVS 3'	AGGATTGGTGACAGAAAA
