# Supplementary material for: Proper chromosome alignment depends on BRCA2 phosphorylation by PLK1
Source: Nat Commun. 2020 Apr 14;11:1819. doi: 10.1038/s41467-020-15689-9 (PMC7156385; doi:10.1038/s41467-020-15689-9)
Supplement: Supplementary file 3 — Description of Additional Supplementary Files [file 41467_2020_15689_MOESM3_ESM.pdf]

Description of Additional Supplementary Files

File Name: Supplementary Movies 1-3
Description: Representative videos of DLD1 stable cells lines bearing BRCA2 WT, BRCA2 S206C and BRCA2- T207A, as indicated.
