# Supplementary material for: Proper chromosome alignment depends on BRCA2 phosphorylation by PLK1
Source: Nat Commun. 2020 Apr 14;11:1819. doi: 10.1038/s41467-020-15689-9 (PMC7156385; doi:10.1038/s41467-020-15689-9)
Supplement: Supplementary file 7 — Reporting Summary [file 41467_2020_15689_MOESM7_ESM.pdf]

nature research

Corresponding author(s): Aura Carreira

Last updated by author(s): Jan 21, 2020

Reporting Summary

Nature Research wishes to improve the reproducibility of the work that we publish. This form provides structure for consistency and transparency in reporting. For further information on Nature Research policies, see Authors & Referees and the Editorial Policy Checklist.

Statistics

For all statistical analyses, confirm that the following items are present in the figure legend, table legend, main text, or Methods section.

n/a	Confirmed	
☐	☒	The exact sample size (*n*) for each experimental group/condition, given as a discrete number and unit of measurement
☐	☒	A statement on whether measurements were taken from distinct samples or whether the same sample was measured repeatedly
☐	☒	The statistical test(s) used AND whether they are one- or two-sided<br>*Only common tests should be described solely by name; describe more complex techniques in the Methods section.*
☒	☐	A description of all covariates tested
☐	☒	A description of any assumptions or corrections, such as tests of normality and adjustment for multiple comparisons
☐	☒	A full description of the statistical parameters including central tendency (e.g. means) or other basic estimates (e.g. regression coefficient) AND variation (e.g. standard deviation) or associated estimates of uncertainty (e.g. confidence intervals)
☐	☒	For null hypothesis testing, the test statistic (e.g. F, t, r) with confidence intervals, effect sizes, degrees of freedom and P value noted<br>*Give P values as exact values whenever suitable.*
☒	☐	For Bayesian analysis, information on the choice of priors and Markov chain Monte Carlo settings
☒	☐	For hierarchical and complex designs, identification of the appropriate level for tests and full reporting of outcomes
☒	☐	Estimates of effect sizes (e.g. Cohen's d, Pearson's r), indicating how they were calculated

Our web collection on statistics for biologists contains articles on many of the points above.

Software and code

Policy information about availability of computer code

Data collection

Radiactive label of the kinase assay was detected by ImageQuant TL Software v8.2.0 (GE Healthcare Life Science). Videomicroscopy images were collected using either a video-microscope (Leica DMI6000) equipped with electron multiplying charge coupled device (EMCCD) or an inverted spinning-disk microscope (Ti-E, Nikon and Yokogawa CSU-X1 spinning head) equipped with sCMOS Hamamatsu Orca Flash 4.0 (pixel size 6.5 µm), both cameras controlled by Metamorph 2.1 software (Molecular Devices). Images were mounted using Image J software (1.51s, NIH). Western blot and Stain-free signals were detected using Image Lab 5.2.1 Software (Bio Rad). NMR data collection was done using Topspin 3.0 Software NMR (Bruker, https://www.bruker.com/products/mr/nmr/nmr-software/software/topspin). ITC data was collected using Origin 7.0 Software (OriginLab). Fluorescence images were collected in an upright Leica DM6000B widefield microscope equipped with a Leica Plan Apo 63x NA 1.4 oil immersion objective. The camera used is a Hamamatsu Flash 4.0 sCMOS controlled with MetaMorph2.1 software (Molecular Devices). For aneuploidy, fluorescence images were collected in an inverted confocal Leica SP5 microscope with a plan Apo 63x NA 1.4 oil immersion objective using the software Leica LAS AF. Flow cytometry data was collected in a BD FACSCanto II (BD Bioscience) using FACSDiva software v6.0. Diffraction data were collected at the Proxima 1 beamline (SOLEIL synchrotron, Gif-sur-Yvette, France).

Data analysis

NMR data was analysed using CcpNmr Analysis 2.4.2 Software http://www.ccpn.ac.uk/v2-software/software/analysis). ITC data was analysed using Origin 7.0 Software (OriginLab). Flow cytometry data was analysed using FlowJo Software version 10.4.2 software (Tree Star Inc). For statistical analysis and graphical data presentation we used Prism (version Mac OS X 8.3.0 (328)) GraphPad Software. Image data was analysed using ImageJ (1.51s NIH, https://imagej.nih.gov). The dataset was indexed and integrated using XDS1 through the autoPROC package. The structure was solved by molecular replacement using PHENIX (Phaser) software. Refinement was performed using BUSTER and PHENIX. The model was built with Coot software. Representation of the structure was performed in Pymol v1.7.4.0 (Schrödinger, LLC).

For manuscripts utilizing custom algorithms or software that are central to the research but not yet described in published literature, software must be made available to editors/reviewers. We strongly encourage code deposition in a community repository (e.g. GitHub). See the Nature Research guidelines for submitting code & software for further information.

Data

Policy information about availability of data

All manuscripts must include a data availability statement. This statement should provide the following information, where applicable:

- Accession codes, unique identifiers, or web links for publicly available datasets
- A list of figures that have associated raw data
- A description of any restrictions on data availability

The PDB coordinates have been deposited to the PDB repository: deposition ID : D_1200010677. They are now available using the PDB code 6GY2

Field-specific reporting

Please select the one below that is the best fit for your research. If you are not sure, read the appropriate sections before making your selection.

☒ Life sciences ☐ Behavioural & social sciences ☐ Ecological, evolutionary & environmental sciences

For a reference copy of the document with all sections, see nature.com/documents/nr-reporting-summary-flat.pdf

Life sciences study design

All studies must disclose on these points even when the disclosure is negative.

Sample size	No sample size calculation was performed. We chose the sample size to obtain a representative number of cells from several independent experiments to get enough statistical power.
Data exclusions	No data has been excluded
Replication	All the experiments were reliably reproduced, the number of independent experiments are specified in the figure legends for all experiments.
Randomization	For all cell based experiments the control and the treated cells are coming from the same populations and treated at the same time; the comparison of different cell lines/clones was performed using similar passage of the cells.
Blinding	All the immunofluorescence and video-microscopy experiments were performed blinded.

Reporting for specific materials, systems and methods

We require information from authors about some types of materials, experimental systems and methods used in many studies. Here, indicate whether each material, system or method listed is relevant to your study. If you are not sure if a list item applies to your research, read the appropriate section before selecting a response.

Materials & experimental systems

n/a	Involved in the study
☐	☒ Antibodies
☐	☒ Eukaryotic cell lines
☒	☐ Palaeontology
☒	☐ Animals and other organisms
☒	☐ Human research participants
☒	☐ Clinical data

Methods

n/a	Involved in the study
☒	☐ ChIP-seq
☐	☒ Flow cytometry
☒	☐ MRI-based neuroimaging

Antibodies

Antibodies used	For WesternBlot: Antibody anti p676-BUBR1 was obtained from Erich A. Nigg laboratory (Elowe et al., 2007). Mouse anti-MBP (1:5000, R29, Cat. #MA5-14122, Thermo Fisher Scientific), mouse anti-BRCA2 (1:1000, OP95, EMD Millipore), rabbit anti-pT207-BRCA2 (raised for this study using the peptide 203TLSS-pT-VLIVRNEEAC as antigen, Genscript) (1:1000), rabbit anti-GFP (1:5000, Protein Expression and Purification Core Facility, Institut Curie), mouse anti-PLK1 (1:5000, clone 35-206, Cat. #05-844, EMD Millipore), mouse anti-BUBR1 (1:1000, Cat. #612502, BD Transduction Laboratories), rabbit anti-BUBR1 (1:2000, Cat. #A300-386A, Bethyl Laboratories), mouse anti-PP2A C subunit (1:1000, clone 1D6, Cat. #05-421, EMD Millipore), rabbit anti-pT680-BUBR1 (1:1000, EPR 19958, Cat. #ab200061). Horseradish peroxidase (HRP) conjugated 2nd antibodies used: mouse-IgGκ BP-HRP (IB: 1:10 000, Cat. #sc-516102, Santa Cruz), goat anti-rabbit IgG-HRP (IB: 1:5000, Cat. #sc-2054, Santa Cruz), goat anti-mouse IgG-HRP (1:10 000, Cat.# 115-035-003, Interchim), goat anti-rabbit IgG-HRP (1:10 000, Interchim, Cat.# 111-035-003). For Immunofluorescence: human anti-CREST (1:100, Cat. #15-234-0001, Antibodies Online), rabbit anti-pT680-BUBR1 (1:500, clone EPR 19958, Abcam, Cat. #ab200061), mouse anti-PLK1 (1:500, clone F-8, Santa Cruz Biotechnology, Cat. #sc-17783), rabbit anti-BRCA2 (1:500, CA1033, EMD Millipore), mouse anti-α-tubulin (1:5000, GT114, Cat. #GTX628802, Euromedex), anti-pSer139-γH2AX (1:1000, clone JBW301, EMD-Millipore, Cat. #05-636), anti-RAD51 (1:100, clone H-92, Santa Cruz Biotechnology, Cat.

	#sc-8349),<br>goat anti-human Alexa-488 (1:1000, Cat. # A11013, Life Technologies), donkey anti-rabbit Alexa-488 (1:1000, Cat. #A-21206, Thermo Fisher Scientific), goat anti-human Alexa-633 (1:500, Cat. # A21091, Life Technologies), donkey anti-mouse Alexa-488 (1:1000, Cat. #A-21202, Thermo Fisher Scientific), donkey anti-mouse Alexa-594 (1:1000, Cat. #A-21203, Thermo Fisher Scientific), goat anti-human Alexa-555 (1:1000, Cat. #A-21433, Thermo Fisher Scientific)<br>This information is described in the Methods section.
Validation	Validations are based on the datasheets from the manufacturers (RRIDs of the antibodies are provided). The specificity of anti pS676 BUBR1 is described in Elowe et al., 2007 Genes and Dev. Anti GFP antibody was made in house from recombinant GFP in rabbit, purified using NHS-EGFP affinity purification column and validated by immunofluorescence in HeLa cells transfected with a Rab6-GFP construct. The pT207-BRCA2 antibody was validated using an ELISA with the pre-immune serum compared to the phospho-specific antibody.

Eukaryotic cell lines

Policy information about cell lines

Cell line source(s)	The BRCA2 deficient colorectal adenocarcinoma cell line DLD1 BRCA2-/- (Hucl, T. et al 2008) (HD 105-007) and the parental cell line DLD1 BRCA2+/+ (HD-PAR-008) was purchased from Horizon Discovery (Cambridge, England). The human cell lines HEK293T and U2OS cells (kind gift from Dr. Mounira Amor-Gueret) (Institut Curie, Orsay, FR) were originally purchased from ATCC. sf9 insect cells come from the Protein Expression and Purification Core Facility, Institut Curie
Authentication	The authentication of all cells was done by genotyping in our Department
Mycoplasma contamination	All cells were tested negative for mycoplasma using standard mycoplasma kit
Commonly misidentified lines<br>(See ICLAC register)	The only commonly misidentified cell-line used in this study is the HEK293T that we only used for protein purifications and the cells have been genotyped.

Flow Cytometry

Plots

Confirm that:

☒ The axis labels state the marker and fluorochrome used (e.g. CD4-FITC).

☒ The axis scales are clearly visible. Include numbers along axes only for bottom left plot of group (a 'group' is an analysis of identical markers).

☒ All plots are contour plots with outliers or pseudocolor plots.

☒ A numerical value for number of cells or percentage (with statistics) is provided.

Methodology

Sample preparation	DLD1 BRCA2-/- cells stably expressing full-length GFPMBP-BRCA2 and the variants (S206C and T207A) were nocodazole (100 ng/ml, Sigma-Aldrich) or mock treated for 14h, trypsinized, washed in PBS and fixed in cold 70% ethanol at -20ºC overnight. The cells were washed twice in cold 1 x PBS before staining with rabbit anti-histone3 (phosphor-Ser10) antibody (1:200, Cat. #06-570, EMD Millipore) in staining buffer (1 x PBS, 3% FBS) for 1h in room temperature. The cells were then washed with 1 x PBS before staining for 30 min at room temperature with a chicken anti-rabbit Alexa-Fluor-647-conjugated antibody (1:250, Cat. #A-21443, Life Technologies). After one wash in 1 x PBS the cells were resuspended in 7-AAD (559925, BD Pharmingen) to stain DNA and thereafter diluted in staining buffer.
Instrument	The DNA content, BrdU and p-Histone 3 staining were visualized with a FACSCanto II (BD Bioscience) using the FACSDiva software.
Software	Data were analysed with FlowJo 10.4.2 software (Tree Star Inc.).
Cell population abundance	10 000-20 000 events of FSC singlets (gated in FSC-W vs FSC-A plot) were collected for each experiment, the whole FSC singlet cell population were analysed for BrdU and p-Histone 3 positive cells and the DNA content.
Gating strategy	All collected cells were plotted in a Forward Scatter (FSC-A) versus Side Scatter (SSC-A) diagram to gate for viable cells based on size and granularity, the gated FSC-A/SSC-A population were displayed in a FSC-W versus FSC-A graph to further exclude doublets. The FSC singlet populations were displayed in a Alexa Fluor 647 (p-histone 3) -A versus DNA 7AAD-A graph for the p-histone-3 analyis and in a BrdU-APC-A versus 7AAD-A plot for the BrdU analysis. The gate for p-histone-3 positive population was based on non-stained Alexa Fluor 647 p-histone 3 cells (cells from the same Nocodazole treatment) and the gate for BrdU positive cells on non-stained BrdU cells. For the cell cycle profiles, the FSC singlet populations were displayed in a DNA 7AAD-A histogram and the gating of the cell cycle phases were based on the intensity of the 7AAD fluorescent signal (1X for G0/G1 and 2x for G2/M).

☒ Tick this box to confirm that a figure exemplifying the gating strategy is provided in the Supplementary Information.
